# Proteomic analysis of rose apple reveals defense proteins activated during *Lasiodiplodia theobromae* infection

**DOI:** 10.1007/s10123-026-00808-1

**Published:** 2026-04-07

**Authors:** Muhammad Naveed Khan, Norasfaliza Rahmad, Nurhaida Kamaruddin, Jameel R. Al-Obaidi

**Affiliations:** 1 Department of Biology, Faculty of Science and Mathematics, Universiti Pendidikan Sultan Idris, Tanjong Malim, Perak, 35900 Malaysia; 2 Agro-Biotechnology Institute Malaysia (ABI), National Institutes of Biotechnology Malaysia (NIBM), c/o MARDI Headquarters, Serdang, Selangor 43400 Malaysia; 3 Applied Science Research Center, Applied Science Private University, Amman, Jordan

**Keywords:** Fruit proteomics, Rose apple, Post-harvest, Plant defence

## Abstract

Rose apple (*Syzygium samarangense*) is a highly nutritious tropical fruit with high economic value, but its post-harvest quality is drastically impaired by fungal pathogens, especially *Lasiodiplodia theobromae*. This experiment used a proteomics method to study the molecular events of the rose apple fruit in response to the infection of *L. theobromae*. The fruits were separated into control and pathogen-inoculated groups, and protein extraction and separation were performed using two-dimensional gel electrophoresis (2-DE). Proteins that showed a difference in expression were determined by MALDI-ToF/ToF MS/MS and further examined by protein-protein interaction network mapping. Fourteen spots were identified that showed significant variation in their expression between the non-infected and the infected samples. Proteins associated with stress, such as Heat shock protein 90 − 1 and Heat shock 70 kDa protein, were significantly increased, suggesting a possible involvement in cell defence mechanisms. Also, there was a higher expression of Peptidyl-prolyl cis-trans isomerase, which may indicate increased protein folding activity during pathogen stress. Conversely, the downregulation was observed in the Linoleate 9 S-lipoxygenase, which suggests inhibition of fatty acid synthesis during infections. All these results demonstrate that there is extensive translational-level reprogramming of stress adaptation and protein homeostasis in response to fungal invasion. The proteins identified represent potential candidates for future studies aimed at understanding resistance mechanisms in rose apple. Their roles need further functional validation to have a better insight into their roles and to facilitate the development of effective postharvest management strategies.

**Supplementary Information:**

The online version contains supplementary material available at 10.1007/s10123-026-00808-1.

## Introduction

Rose apple (*Syzygium samarangense*), widely known as wax apple, is one of the important tropical fruit that are widely grown in most regions of the world and its juicy, crisp and slightly sweet fruits are used both fresh and processed (Banadka et al. 2022). The rose apple fruits are usually pear shaped and can measure between 3.4 and 6 cm long and 4 to 5 cm wide with a weight of between 70 and 200 g (Subbulakshmi et al.  2021) The fruits contain significant nutrition value and are rich in water, carbohydrates, moderate levels of protein, essential minerals, vitamins A and C, dietary fibre, and various antioxidant phytochemicals, such as phenolic compounds, flavonoids (Mukaromah 2020). In addition to culinary purposes, rose apple has been traditionally used to treat a wide range of health-related disorders (Saroj and Shah 2023). It exhibited antimicrobial and anti- inflammatory properties (Salem et al. 2023). In addition, rose apple is also used in different value-added products, including candies, jam, juices, and syrups (Majumder et al. 2021). Due to such a set of nutritional advantages and positive sensory characteristics, rose apple has been noticed as a new fruit in the market of most countries and can be found both in its fresh and processed versions (Majumder et al. 2021). Small-scale farmers produce the crop predominantly in Malaysia, which is now seen as a high-value horticultural product both in the domestic and export market, with an estimated yearly production of about 9 million US dollars and a market price of about 3 USD per kilogram (Al-Obaidi et al. 2023). Nevertheless, due to the high level of moisture, the presence of a lot of nutrients and the sensitive skin, rose apple fruits are especially susceptible to infection by microorganisms, especially fungi, both before and after harvest (Meng et al. 2021). These fruits are not resistant to extended storage or long-distance transportation (Esua et al. 2017). Postharvest fungal diseases in Malaysia are approximated to lead to about one-third of annual losses associated with rose apple production, which is a significant limitation to growers and exporters (Al-Obaidi et al. 2023). Postharvest diseases lower yield and quality, and the losses incurred during storage, transport, and marketing are highly dependent on postharvest handling and packaging (Bano et al. 2023). Mechanical damage of the fruit surface, including cracks, abrasions, cuts, and insect punctures and other wounds during harvest and handling, constitute valuable points of entry of pathogens, and allow the rapid development of the disease in the storage (Singh and Sharma 2018). Direct penetration through the cuticle or exploitation of natural openings on the surface of the fruit can also be used by some fungi (Fenta et al. 2023). *Lasiodiplodia theobromae* is another pathogen that has lately been discovered to cause postharvest disease in Malaysia as far as rose apple fruit rot is concerned (Khan et al. 2025). The initial defence mechanisms of the fruit include expression of proteins and enzymes that help to protect against fungal attack, and knowledge of these molecular processes is critical in developing effective control measures of fungal attack on the postharvest diseases (Zhang et al. 2023). The course of the fungal infection is strictly interconnected with the physiological age of the fruit, the mechanical damage, and the storage conditions including the temperature and humidity in the storage environment (Sui et al. 2025). All these reasons make it important to thoroughly study the problem of postharvest diseases in rose apple, especially taking into consideration the new fungal threats that pose a threat to the supply of fruits and grower profits. When plants are confronted by pathogenic organisms, they activate a complex immune system (Jian et al. 2024). The plant cell wall represents the first physical barrier against invading pathogens. However, successful pathogens such as *Lasiodiplodia theobromae* are capable of overcoming this barrier through the secretion of cell wall–degrading enzymes and virulence-associated metabolites, facilitating host tissue colonization (Mohapatra et al. 2025). In response to infection, host plants activate defense-related pathways involving stress-associated proteins, antioxidant systems, and metabolic adjustments aimed at restricting pathogen spread (Peng et al. 2022). These defense responses are often accompanied by changes in proteins associated with stress signaling, energy metabolism, and protein folding, reflecting the plant’s attempt to cope with pathogen-induced stress (Chen et al. 2026). Pathogens cause this defense to be overcome by releasing effector molecules, which are then sensed by plant resistance (R) proteins due to perceiving pathogen-derived avirulence (Avr) factors, triggering effector-triggered immunity (ETI), a more specific and more powerful defense mechanism (Xing et al. 2023).

In this regard, there is a high demand for strategies that preserve the quality of fruits during transportation and storage and reduce postharvest disease losses. Proteins are the main functional molecules in the cells that promote vital biological processes, including enzymatic reactions, signal transduction pathways, structural maintenance, and molecular interactions (Gobena et al. 2024). As such, proteomics has emerged as a major field of study in the discovery of molecular processes involved in normal physiological processes as well as pathological processes (Chandramouli and Qian 2009). Proteomics can be defined as the overall study of proteins that are found within a biological system at a given time (Jiang et al. 2024). Proteome as the total amount of proteins expressed by a biological entity, is extremely dynamic and constantly changes in relation to internal and external factors (Aslam et al. 2017). It is not only protein identification and functional characterization but also quantification of protein levels, study of post-translational modifications, protein-protein interaction, and subcellular localization (Ortea et al. 2016). It has wide applications and enables protein expression profiling, the measurement of changes in protein abundance, protein interaction mapping, the establishment of complex interaction networks between proteins (Jiang et al. 2025). Protein expression profiling comes in handy especially when it comes to the detection of differentially expressed proteins to environmental signals or stressors. As an example, alterations in the patterns of protein expression in plants during pathogen invasion can be utilized to identify proteins that mediate stress-tolerance and immune defense (Domingo et al. 2024). In this study proteomic can provide a strong method of molecular analysis of the interaction between rose apple and *Lasiodiplodia* spp by comparing the protein homeostasis of infected fruits and healthy controls. This study aims to address the current lack of proteomic information describing the molecular responses of rose apple fruit during infection by *Lasiodiplodia theobromae*. By characterizing the proteomic profiles of infected and non-infected postharvest fruits, this work seeks to provide initial insights into the host proteins and molecular pathways potentially involved in the rose apple defence response to fungal infection.

## Materials and methods

### Sample collection

In Malaysia, the local rose apple fruits were sourced postharvest in one of the commercial fruit markets at Perak (4°35′ N, 101°05′ E), at the point when the skin colour satisfied the market requirements, and this was about 50 days after flowering. Fruits of uniform size (approximately 4–6 cm in length and 70–200 g in weight) with smooth, intact skin and characteristic market-mature coloration (light pink to reddish surface) were randomly selected, while fruits showing visible injuries, decay, or other defects were excluded. A total of 20 physiologically mature fruits were selected and randomly divided into two experimental groups: control and *L. theobromae*-inoculated treatments (10 fruits per group). Each treatment consisted of four biological replicates, with individual fruits serving as independent experimental units for proteomic analysis. Fruits were washed and sanitized using 70% ethanol and graded on the basis of size and then stored at room temperature till used in the experiment.

### Fungal cultivation, preparation of inoculum, and spore germination assay

**Fig. 1:**
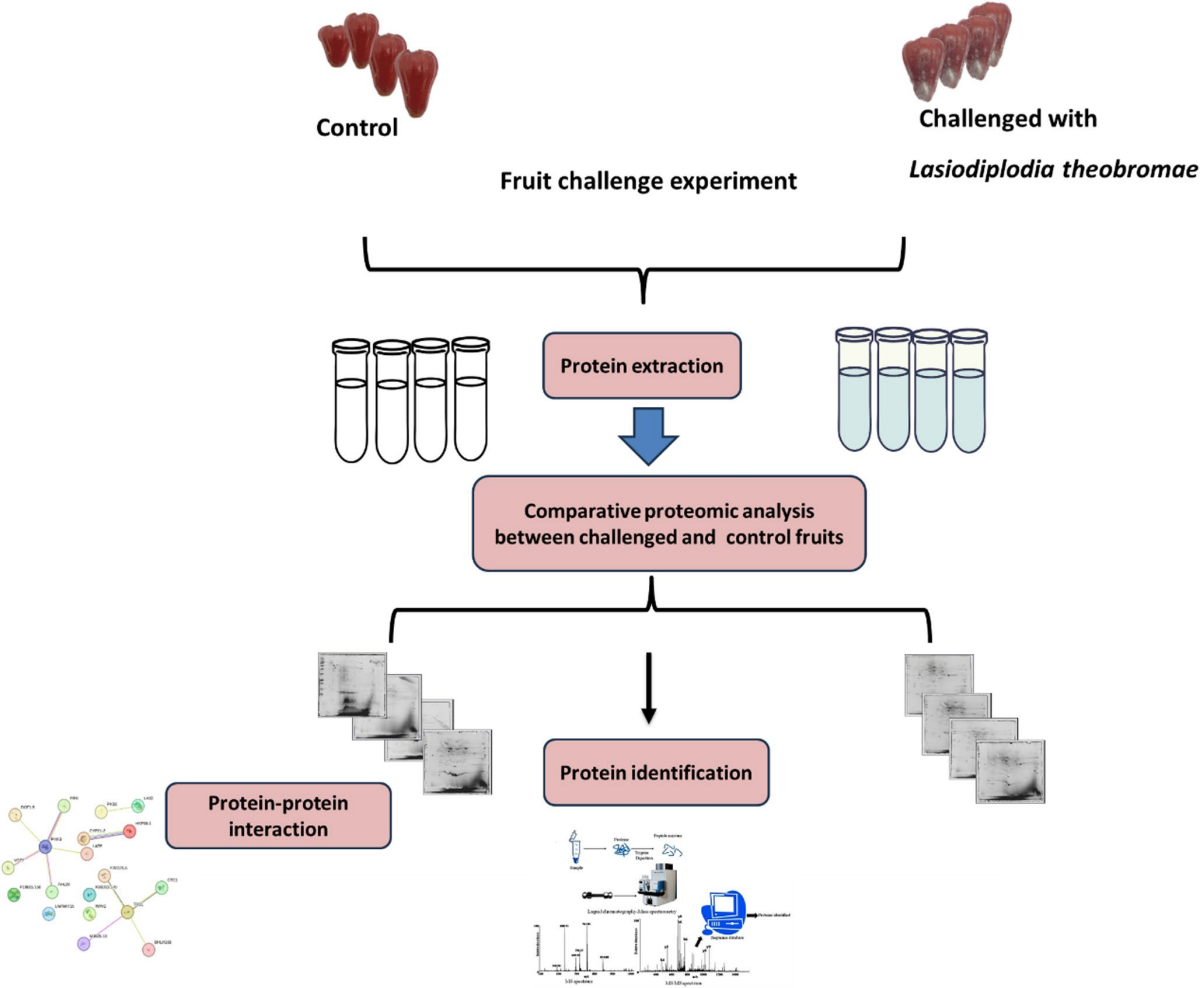
Schematic overview of the experimental workflow used for proteomic profiling of *Lasiodiplodia*-infected rose apple fruits. Two experimental sets were established, consisting of non-inoculated control fruits and fruits challenged with *Lasiodiplodia*. Following inoculation, total proteins were isolated and resolved using two-dimensional gel electrophoresis (2-DE). Protein spots were subsequently detected and identified by MALDI mass spectrometry. In addition, protein–protein interaction analysis was performed to elucidate the molecular responses of rose apple fruit to fungal infection

The selected isolate was previously identified and deposited as ITS (PQ584813) and TEF1-α (PV155110) in Sterling liquid medium using a (1 × 10⁶ conidia/mL) suspension of *L. theobromae*. A longitudinal axis was drawn on each of ten fruits, and two pinprick wounds were created on the longitudinal axis. An aliquot (10 µL) of the conidial suspension was carefully applied to the wounded area. The control group was made up of ten fruits, which were treated in the same way but inoculated with sterile distilled water. Each of the fruits was put in the plastic boxes with the moistened filter paper and left to incubate at the temperatures of 27 °C and the relative humidity of 90 ± 10%. The earliest visible symptoms of infection were observed at 96 h after inoculation, and this time point was therefore selected for proteomic analysis as it represents the initial stage of visible host–pathogen interaction in rose apple fruit under our experimental conditions. Proteins were subsequently isolated using control and inoculated fruits and resolved using two-dimensional gel electrophoresis (2-DE) following incubation to compare the protein expression patterns. Protein spots were subsequently subjected to MALDI -ToF/ToF MS/MS, which facilitated the detection of proteins showing significant changes in expression levels and the establishment of protein-protein interaction networks of the chosen protein spots. A summary of the working process in the experiment is given in Fig. 1.

### Protein extraction

Proteins were extracted from frozen rose apple fruit tissues (5 × 5 × 5 mm cubes) from both *Lasiodiplodia*-infected and control samples using a phenol/ammonium acetate precipitation procedure adapted from previously reported protocols. Briefly, frozen tissues were finely ground to a powder in a pre-chilled mortar and pestle under liquid nitrogen. The resulting powder was immediately suspended in 1 mL of Tris-buffered phenol (pH 8.8) and thoroughly mixed with an equal volume (1 mL) of extraction buffer containing 0.9% sucrose, 50 mM Tris–HCl (pH 6.8), 10 mM EDTA and 0.4% β-mercaptoethanol.

The mixture was gently homogenized and centrifuged at 6000 rpm for 10 min to allow phase separation. The upper phenolic phase was carefully collected and subjected to a second extraction using an equal volume of fresh extraction buffer and phenol. Following centrifugation, the phenolic layer was recovered and proteins were precipitated by adding five volumes of ice-cold 0.1 M ammonium acetate prepared in methanol. The samples were incubated at − 20 °C for 24 h to facilitate protein precipitation.

After incubation, the samples were centrifuged for 10 min and the resulting protein pellets were washed twice with 0.1 M ammonium acetate in absolute methanol (20 mL), followed by two additional washes with ice-cold 80% acetone. The pellets were then air-dried and subsequently dissolved in 0.2 mL of protein solubilisation buffer containing 7 M urea, 2 M thiourea, 4% CHAPS and 0.002% bromophenol blue.

### 2DE and image analyses

Isoelectric focusing (IEF) followed by two-dimensional difference gel electrophoresis (2-DE) (four biological replicates) was performed to resolve proteins previously solubilised in rehydration buffer containing 7 M urea, 2 M thiourea, 2% CHAPS, 0.5% immobilised pH gradient (IPG 4–7), and 0.0025% bromophenol blue. For the first-dimension separation, a total of 300 µg of protein was diluted in rehydration buffer and passively rehydrated onto 13 cm IPG strips (pH 4–7; GE Healthcare) for 24 h.

Isoelectric focusing was subsequently carried out using an Ettan IPGphor system (GE Healthcare) according to the focusing programme described in previous studies. After focusing, the IPG strips were subjected to two sequential equilibration steps prior to second-dimension separation. The strips were then placed on 12% SDS–polyacrylamide gels prepared using approximately 50 mL gel solution per gel and electrophoresed using an SE600 vertical electrophoresis system (GE Healthcare) at a constant current of 15 mA per gel for 300 min.

Following electrophoresis, gels were stained with colloidal Coomassie Brilliant Blue G-250 and scanned using a Bio-Rad densitometry system. Gel image processing and spot quantification were carried out with Progenesis SameSpots software (Nonlinear Dynamics, Durham, NC, USA) using four biological replicates per treatment. Only protein spots exhibiting statistically significant abundance changes based on Student’s t-test (*p* < 0.05) were selected for subsequent protein identification.

### Protein gel digestion and MS/MS analysis

Manual excision of protein spots in 2DE gel analysis followed by in-gel trypsin digestion according to Al-Obaidi and his colleagues, with some modifications (Al-Obaidi et al. 2017). Peptide mixtures obtained after enzymatic digestion were purified and concentrated using C18 ZipTip pipette tips (Millipore, Bedford, MA, USA) prior to mass spectrometric analysis. The cleaned peptides were analysed by MALDI-TOF/TOF mass spectrometry using an ultrafleXtreme instrument (Bruker Daltonics). Laser desorption and ionisation were achieved with a 355-nm Nd: YAG laser. Ion acceleration was performed using a 25-kV extraction voltage, and spectra were generated by accumulating signals from 1000 laser shots per spectrum. Data acquisition was conducted in positive reflectron mode at 1 kV with a mass tolerance of 20 ppm. Database searching and peptide identification were performed using the Mascot search engine (Matrix Science, UK) against the Swiss-Prot and NCBI protein databases, with taxonomy restricted to Viridiplantae. Search parameters included trypsin as the digestion enzyme with one missed cleavage allowed, carbamidomethylation of cysteine as a fixed modification, and oxidation of methionine as a variable modification, using monoisotopic mass values. Protein identifications were considered significant when the Mascot score exceeded the significance threshold, and peptide matches were filtered using a false discovery rate (FDR) threshold of 1%0.2.6 Clustered heatmap of differentially abundant proteins.

The important proteins were grouped and visualized in a heat map to show the abundance profile of the modified proteins. The patterns of abundance of rose apple proteins were different when comparing the challenge experiment to the control fruits.

### Protein-protein interaction

Protein–protein interaction (PPI) analysis was performed using the STRING platform (accessed on 24 March 2025) to explore possible functional associations among the proteins of interest. Only interactions within the top 0.4 fraction of STRING confidence scores were retained in order to achieve an appropriate balance between detection sensitivity and interaction reliability. The interaction network integrates multiple evidence channels, including experimentally validated interactions, curated pathway databases (e.g., IntAct and MINT), and computationally inferred predictions. *Arabidopsis thaliana* was used as the reference species for network construction due to the absence of a protein–protein interaction database for *Syzygium samarangense*. Therefore, the resulting interaction networks represent predicted functional associations based on homologous proteins.

## Results

Protein abundance profiles of rose apple fruits subjected to *Lasiodiplodia* infection and non-infected controls were examined using two-dimensional gel electrophoresis (2-DE). For each experimental group, four independent biological replicates were analysed (Supplementary Fig. 1). Minor gel distortions were observed; however, only consistently detected and accurately matched spots across all biological replicates were considered for quantitative analysis. Overall, 32 reproducible protein spots were detected across the gels. Among these, 14 protein spots showed statistically significant abundance differences between infected and control samples, based on a fold change threshold greater than 1.5 and a significance level of *p* < 0.05.

**Fig. 2:**
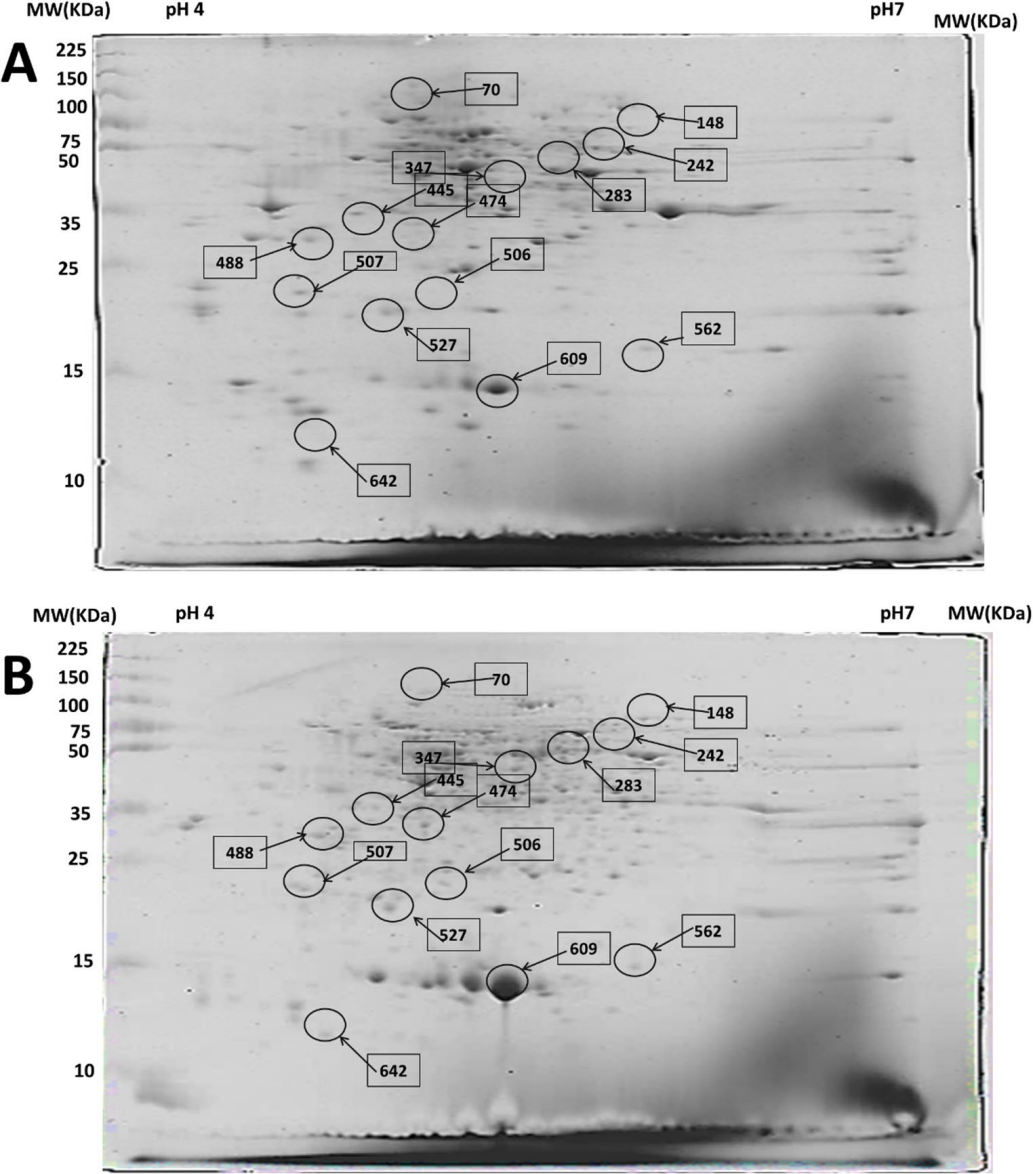
Representative two-dimensional electrophoresis (2-DE) gels showing protein profiles of control (**a**) and *Lasiodiplodia***-**infected (**b**) rose apple fruits. Protein separation was based on isoelectric point (pI) and molecular mass (MW). The numbered spots (70, 148, 242, 283, 347, 445, 474, 488, 506, 527, 562, 609, 648, and 649) represent proteins identified by MALDI-ToF/ToF MS/MS that showed significant abundance changes between control and infected samples. Functional annotation suggests these proteins are mainly associated with stress responses, lipid metabolism, and ribosomal functions

A representative gel image and corresponding spot analysis for each condition are shown in Fig. 2, with protein spots annotated according to their assigned identification numbers. Of the 14 differentially accumulated proteins, seven were up-regulated, whereas seven were down-regulated in response to *Lasiodiplodia* infection relative to the control group. The master gel displayed in Fig. 2 highlights all significantly altered protein spots detected in both treatments and provides an integrated overview of the 2-DE profiles of control and infected rose apple fruits. The positions of the labelled spots reflect their estimated molecular weights and isoelectric points (pI), enabling direct visual comparison of proteomic alterations associated with fungal challenge.

**Fig. 3:**
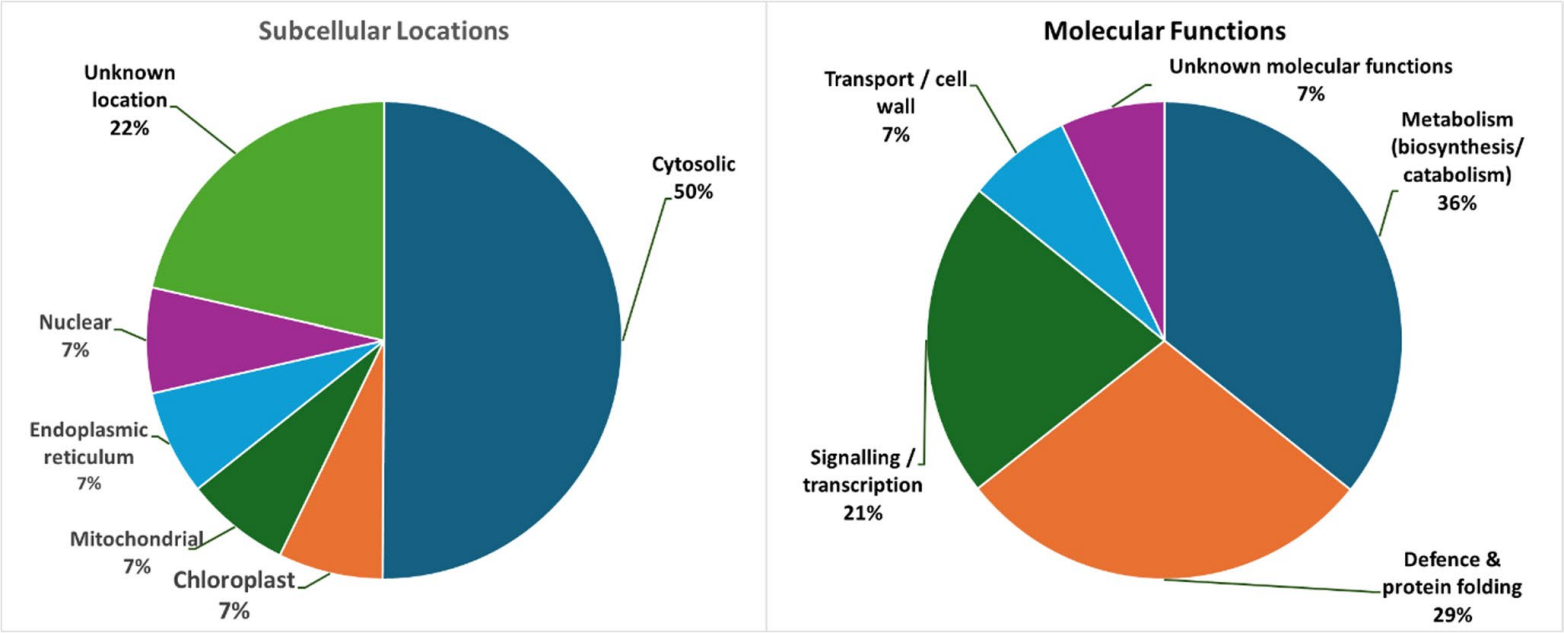
Protein classification by cellular compartment (left) and molecular function (right), with chart segments showing the percentage assigned to each class

MALDI-ToF/ToF MS/MS analysis enabled the successful identification of 14 proteins corresponding to the 14 selected protein spots (Tables 1 and 2). Several proteins displayed increased abundance in the *Lasiodiplodia*-challenged samples compared with the control fruits, including heat shock protein 90 − 1 (spot 70), peptidyl-prolyl cis–trans isomerase (spot 148) and heat shock 70 kDa protein (spot 648). Functional annotation and subcellular localisation of the identified rose apple proteins are summarised in Fig. 3. A major percentage of the proteins identified belonged to the cytosolic category, with 50% of the proteins located in the cytosol. Another 29% of the proteins were organelle-associated, such as the chloroplast, mitochondria, endoplasmic reticulum, and nucleus (with each having approximately 7% of the total). The other 21% of proteins were uncertain or had multiple possible localization sites and were thus categorized as having unknown subcellular location. Molecular functional proteins were approximately 36% of the proteins, with defence and protein folding-related proteins (including heat shock proteins, and peptidylprolyl cis-trans isomerase) approximately 29%. Signalling and transcription-related proteins accounted 21%, transport or cell-wall-associated proteins are 7%, and around 7% of the proteins were classified as having unknown or poorly characterized molecular functions. These observations suggest that proteins located in the cytoplasm and cellular organelles may participate in the defence response against *Lasiodiplodia.* Furthermore, the relatively high proportion of proteins lacking functional annotation highlights the necessity for additional studies to elucidate their biological roles in rose apple fruit physiology, especially under postharvest fungal challenge conditions.

**Table 1:** Identification of differentially expressed upregulated proteins in *Lasiodiplodia*-challenged rose apple fruit using MALDI-ToF/ToF MS/MS analysis

Spot ID	Identified protein	Reference species	Theoretical MW(KDa)/pI*	Experimental Mw(KDa)/PI	Identification score	Functional annotation	Fold change	*P*- value	*Expression value* Control Infected	
70	Heat shock protein 90 − 1	*Arabidopsis* *thaliana*	80635.46/4.96	85/5.02	77	Plant defense/stress response	1.5	0.043	8.03E + 05	1.22E + 06
148	Peptidyl-prolyl cis-trans isomerase	*Arabidopsis thaliana*	24506.16/6.52	61/6.32	46	Protien folding	1.8	0.001	4.77E + 05	8.37E + 05
242	Telomere repeat-binding factor 1	*Arabidopsis* *thaliana*	39625.99/6.96	47/7.5	56	Transcription regulation	1.6	0.014	2.70E + 05	4.29E + 05
283	Alpha/beta hydrolase domain-containing protein WAV2	*Arabidopsis thaliana*	34341.17/9.10	43/6.85	47	Proteolysis	1.7	0.026	4.92E + 05	8.17E + 05
347	Probable 3- hydroxyisobutyrate dehydrogenase-like 1, mitochondrial	*Arabidopsis thaliana*	35371.76/8.58	39/6.03	47	Branched chain amino acid catabolism	1.5	0.043	5.85E + 05	8.89E + 05
648	Heat shock 70 kDa protein	*Zea mays*	70573.2/5.22	74/5.2	68	Stress response	1.7	0.021	1.78E + 06	3.07E + 06
649	Trypsin inhibitor 4	*Cucurbita maxima*	3669.32/6.05	40/7.26	48	Unknown	1.6	0.044	1.59E + 06	2.46E + 06

**Table 2:** Identification of differentially expressed downregulated proteins in *Lasiodiplodia*-challenged rose apple fruit using MALDI-ToF/ToF MS/MS analysis

Spot ID	Identified protein	Reference species	Theoretical MW(KDa)/pI*	Experimental Mw(KDa)/PI	Identification score	Functional annotation	Fold change	*P*- value	*Expression value* Control Infected	
474	Scoulerine-9-O- methyltransferase 1	Papaver somniferum	42699.28/5.48	25/5.67	50	Alkaloid metobolism	1.6	0.029	3.52E + 05	2.24E + 05
445	Lanosterol synthase	*Arabidopsis* *thaliana*	86517.67/6.52	29/5.5	45	Triterpinoid biosynthetic process	1.8	0.002	1.39E + 05	7.92E + 04
488	F-box/FBD/LRR-repeat protein At3g51530	*Arabidopsis thaliana*	52751.1/8.73	25/5.11	48	Unknown	1.5	0.01	1.02E + 05	6.85E + 04
506	Linoleate 9 S-lipoxygenase	*Phaseolus* *vulgaris*	84196.8/6.14	24/5.64	48	Fatty acid biosynthesis	1.7	0.022	2.74E + 05	1.62E + 05
527	3-phosphoshikimate 1- carboxyvinyl transferase 1, chloroplastic	*Nicotiana tabacum*	55711.29/8.45	23/5.37	45	Amino-acid biosynthesis	1.8	0.003	5.05E + 05	2.79E + 05
562	WAT1-related protein At5g13670	*Arabidopsis thaliana*	41468.73/9.67	21/7.08	40	Unknown	1.6	0.008	6.11E + 04	3.78E + 04
609	Phytochrome B	*Arabidopsis thaliana*	129331.37/5.6 2	19/6.21	53	Phytochrome signaling pathway	1.6	0.022	2.57E + 07	1.57E + 07

**Fig. 4:**
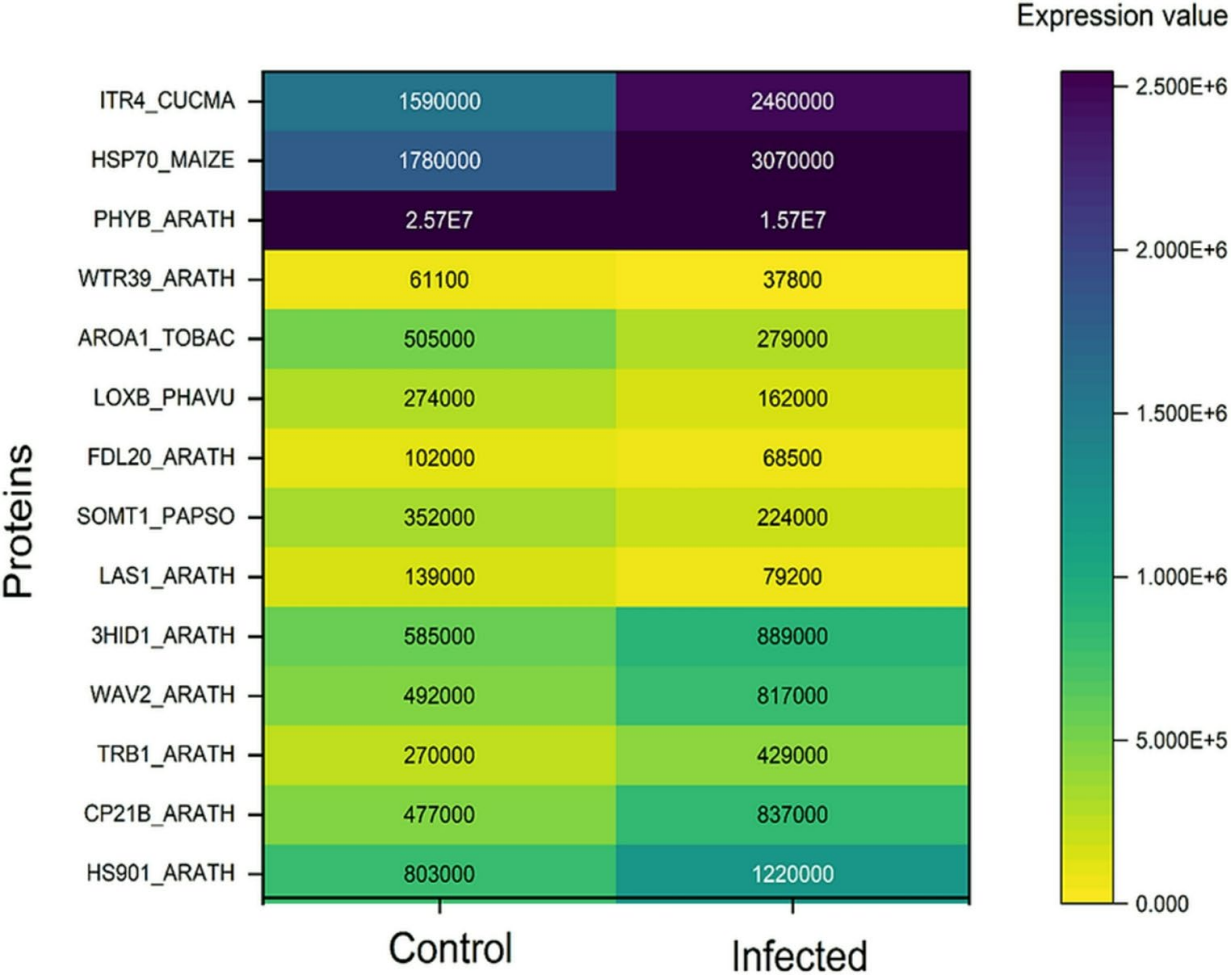
Heatmap illustrating the identified spot volume intensities of altered abundance proteins in rose apple fruit, showing a comparative profile between control and pathogen-infected samples

The normalized volume of spots in the control group and infected group of rose apple fruits challenged with *L. theobromae* was used to create a heatmap (Fig. 4) to show the differentiation of accumulation of major proteins in the fruits. This heatmap shows proteins that are either upregulated or downregulated in infected fruit with blue and yellow hues, respectively, which gives a visual depiction of proteomic alterations associated with pathogen infection.

Significant increases in the levels of Heat shock protein 90 − 1 (Hsp90-1; Spot 70) and Heat shock 70 kDa protein (Hsp70; Spot 648) were observed in infected tissues, which are known to play key roles in plant-pathogen interactions, and thermal or proteotoxic stress responses, which contribute to defence signalling. Peptidyl -prolyl cis -trans isomerase (PPIase; Spot 148), a protein that is involved in protein folding, was also increased, indicating that there is a higher requirement to properly fold defence-related proteins and repair enzymes during infection. High levels of Trypsin inhibitor 4 (Spot 649) may indicate an active defense against fungal proteins and thus defense against host cell walls and tissues. The telomere repeat-binding factor 1 (Spot 242) and Alpha/beta hydrolase domain-containing protein WAV2 (Spot 283) were also found to be up-regulated, which suggests a possible involvement of these proteins in the processes of stress-responsive gene regulation and the adjustment of cell wall properties or hormone-related signalling in the defence response, respectively. Higher levels of the mitochondrial enzyme Probable 3-hydroxyisobutyrate dehydrogenase-like 1 (Spot 347) can indicate a metabolic transition to amino acid catabolism to aid in the production of ATP in response to infection stress.

Conversely, proteins such as linoleate 9 S-lipoxygenase (Spot 506) and lanosterol synthase (Spot 445) were downregulated in infected fruits. This pattern may suggest potential alterations in oxylipin-related signalling and sterol metabolism during disease progression. Similarly, the reduced abundance of Scoulerine 9-O-methyltransferase 1 (Spot 474), an enzyme associated with benzylisoquinoline alkaloid biosynthesis, may indicate possible changes in secondary metabolic pathways during pathogen infection. The decreased levels of 9 S-lipoxygenase (Spot 506) could also reflect a modification of host signalling responses during *L. theobromae* infection or as a consequence of tissue damage associated with disease development. The lower expression of Lanosterol synthase (Spot 445) is in line with the impaired production of sterols and the impaired repair of membranes, whereas the lower expression of WAT1 (Spot 562), a protein that is needed in the secondary cell wall formation and transporting of auxin, indicates a poor ability to strengthen the structural barriers. Equally, the shikimate pathway enzyme 3-phosphoshikimate 1-carboxyvinyltransferase 1 (EPSP synthase; Spot 527) showed reduced abundance in infected samples, which may reflect potential alterations in shikimate pathway metabolism during infection, a pathway that has been associated with the production of several defence-related metabolites in plants. The reduction in Phytochrome B (Spot 609), a light receptor that combines light and defence signalling, a reduced abundance was also observed for the F-box/FBD/LRR repeat protein At3g51530 (Spot 488), a component associated with ubiquitin-mediated protein degradation. This change may reflect potential alterations in protein turnover and signalling processes during infection. Overall, these patterns suggest possible shifts in defence-related pathways during prolonged infection, although further functional validation would be required to confirm these roles. Taken together, the heatmap highlights organized coordination of proteins that are engaged in cellular lipid metabolic activities, redox homeostasis and protein synthesis during the rose apple response to *Lasiodiplodia* infection.

**Fig. 5:**
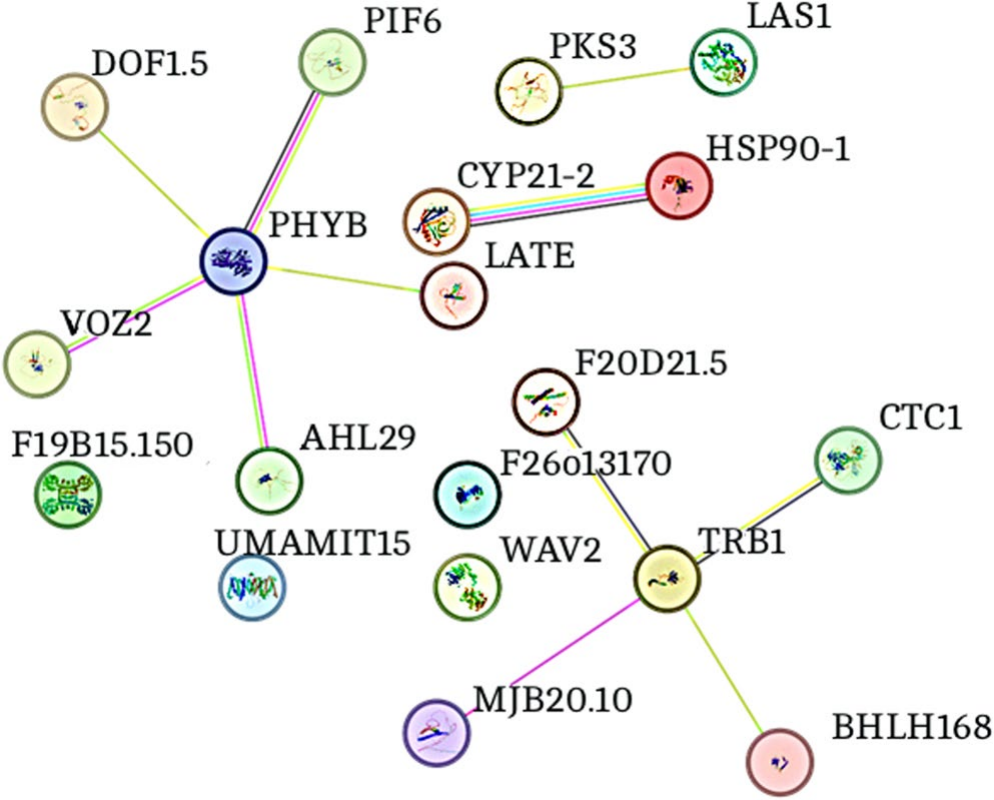
Interaction map of the representative proteins linked with the defence response for the Rose apple infected by *L. theobromae*. Components represented on the network are Heat shock protein 90 − 1, Peptidyl-prolyl cis-trans isomerase, Telomere repeat-binding factor 1, Probable 3-hydroxyisobutyrate dehydrogenase-like 1, mitochondrial, Lanosterol synthase, F-box/FBD/LRR-repeat protein At3g51530, WAT1-related protein At5g13670, Phytochrome B, Heat shock 70 kDa protein, and Trypsin inhibitor

In order to investigate further the functional relationships among these proteins, a PPI network was built with STRING (Fig. 5). The network demonstrated a complex web of interaction involving proteins that are involved in stress responses, metabolism adaptations and defence responses, thus establishing functional protein modules that are related to the response of rose apple fruits to *L. theobromae*. The network hubs were central nodes (Hsp90 -1, Hsp70, PPIase, Telomere repeat -binding factor 1 and Trypsin inhibitor 4), suggesting that these proteins are central nodes in defence response. The location of Hsp90 and Hsp70 in the middle of the picture agrees with the functions of molecular chaperones described to stabilize resistance (R) proteins and mediate recognition of effectors in plant immunity. Their cooperation with PPIase implies the improved quality control system which assists to preserve the functional conformation of defence-related proteins in the conditions of proteotoxic stress.

Moreover, the network brings out a possible connection between physical defense and signalling. An example of such a protein is Alpha/beta hydrolase domain containing protein WAV2, which is involved in root development and stress response and is present here indicating a possible role in strengthening cell wall barriers or controlling hormonal signaling. At the same time, the presence of Trypsin inhibitor 4 suggests the direct biochemical retaliation, when the host produces protease inhibitors to counter fungal hydrolytic enzymes trying to break the plant cell wall. Overall, these findings provide deeper insight into the proteomic landscape associated with infection and reveal both the strengths and limitations of the plant defence response.

## Discussion

The proteomic study provided important information on the molecular-based responses of rose apple fruit to *L. theobromae* challenge. The proteins identified were linked to key biological processes which form the basis of host defence, and the analysis showed a variety of different response patterns. These included the enhancement of protein synthesis, the stress-related pathways, the regulation of lipid metabolism, and the redox homeostasis. It is worth noting that in response to the fungal pathogen, some proteins in turn were very much upregulated, and others were downregulated simultaneously. This wavering expression pattern shows that the rose apple fruit has a complex and multi-dimensional system of defence against fungal invasion. Thus, proteomic data can shed more light on the intracellular processes to explain the observed disease development in the identified period of the experiment.

### Confirmation of the causal pathogen (*L. theobromae*)

The fungal isolate used in this study corresponded to *L. theobromae*, which was previously identified and characterized based on morphological and molecular analyses (He et al. 2025). Pathogenicity tests confirmed its virulence: inoculated rose apple fruits developed distinct dark lesions typical of fungal infection, whereas control fruits remained healthy. These observations confirm *L. theobromae* as a primary postharvest pathogen of rose apple fruit and are consistent with earlier reports of this fungus causing similar diseases in other crops (Chen et al. 2024).

### Physiological and proteomic responses to infection

*L. theobromae* infection caused significant physiological modifications in the rose apple fruit, which were most evident by the high concentration of total proteins in inoculated fruits relative to controls. This is probably the de novo synthesis of defense-related proteins, including pathogenesis-related (PR) proteins and antioxidant enzymes, which are stimulated to limit the colonization of fungi (Chan 2012),(Xu et al. 2022). This trend is consistent with the overall plant stress response, where the accumulation of stress-responsive proteins, chaperones and defense enzymes occurs to curb damage to tissues (Zhang et al. 2020). A similar accumulation of stress-responsive proteins, including chaperones, defense-related enzymes, and proteins associated with energy metabolism, has also been reported in proteomic studies of cashew stems infected with *L. theobromae*, where significant changes in proteins involved in stress responses and cellular signaling were observed during pathogen challenge (Cipriano et al. 2015).

To further define this reaction, 2-DE was followed by MALDI-TOF/TOF mass spectrometry, which detected 14 differentially expressed proteins that were engaged in a complicated host-pathogen interaction. Recent proteomic research has highlighted the dual nature of such proteins: some are increased to limit the growth of pathogens, and others in primary metabolism (e.g., photosynthesis, energy production) are usually decreased to allow the allocation of defenses (Han and Schneiter 2024). As an example, the expression of proteins related to the tricarboxylic acid (TCA) cycle and glycolysis was highly repressed in the *Penicillium expansum*-infected apple fruit, and this is the sign of a trade-off between cellular maintenance and active defense (Xu et al. 2022). Similarly, the defense proteins like PR10 were not only expressed upon infection with *B. cinerea* but also post-translationally modified (phosphorylation, ubiquitination) to increase stability and antifungal efficacy (Li et al. 2023). On the other hand, some pathogens can also cause PR-type proteins, which can mimic host defenses to reduce immunity, which complicates the host-pathogen interaction further (Han and Schneiter 2024).

The two-dimensional electrophoresis (2-DE) in combination with the matrix-assisted laser desorption/ionization time-of-flight/time-of-flight (MALDI-TOF/TOF) mass spectrometry therefore, allowed a comprehensive perspective of the defense architecture in rose apple fruit with 14 differentially expressed proteins that indicate a multifaceted molecular battlefield between the host and the pathogen. Other fruit-pathogen systems have demonstrated similar mechanisms in response to a range of dynamic controls of proteins that regulate reactive oxygen species (ROS) homeostasis, secondary metabolite biosynthesis, hormone signaling, and post-translational alterations that include phosphorylation and ubiquitination that fine-tune immune responses.

### Proteomic changes in rose apple fruits in response to *L. theobromae* infection

The comparative proteomic profiling of rose apple fruits infected by *L. theobromae* showed that a specific molecular signature was composed of 14 differentially expressed proteins. A combination of these proteins leads to the coordination of a complex defense program, which is manifested by changed stress responses, protein folding capacity, and bioenergetic metabolism (Uranga et al. 2017). The high total protein level in the infected tissues argues in favor of the idea that *L. theobromae* is a potent trigger of the physiological apparatus of the host. This accumulation of proteins is probably the host response to counteract the fungal virulence factors, such as cell wall-degrading enzymes (CWDEs) and effector proteins, including LtGAPR1, which can both regulate host ROS levels and suppress host immunity. Subsequently, the fruit tissue seems to counter this by initiating a kind of molecular siege by increasing defense signal transduction and strengthening cell walls, just like in other woody plant-pathogen systems, glucose metabolism and phenylpropanoid biosynthesis are re-programmed to aid defense (Gao et al. 2016).

#### Stress response proteins and heat shock–related pathways

Heat Shock Protein 90 − 1 (Spot 70) and Heat Shock 70 kDa Protein (Spot 648) were significantly increased in the infected fruits in comparison to controls. Even though HSPs were initially linked to thermal stress, it is increasingly becoming clear that the HSP70 and HSP90 families also contribute to pathogen-associated molecular pattern (PAMP)-induced immunity (PTI) in which they maintain the stability of Resistance (R) proteins and signal transduction by kinases that recognize the presence of pathogens (Wei et al. 2021). The high induction observed in this study may reflect proteotoxic stress in rose apple cells and suggests a possible role in maintaining protein stability during pathogen infection. Other host-pathogen systems have also exhibited similar trends: resistant peach cultivars infected with *Monilinia fructicola* demonstrated an enhanced expression of HSP70, which correlated the activity of chaperones with resistance (Papavasileiou et al. 2020); tomatoes infected with *B. cinerea* demonstrated the activity of HSP90 as a part of the response to oxidative stress and aggregates formation during the attack by *necrotrophs* (Shah et al. 2012). Similarly, HSP70 was found to be modulated in citrus plants infected with *Candidatus Liberibacter asiaticus* to preserve protein homeostasis when exposed to biotic stress.

In line with this quality control of the protein, Peptidyl-prolyl cis-trans isomerase (Spot 148) (cyclophilin) was also increased. The enzyme is involved in cistrans isomerization of peptide bonds, which hastens protein folding (Kaur et al. 2015). Its induction implies an increased requirement for efficient folding of damaged or recently synthesized defense proteins, which is not uncommon in wheat infected with *Puccinia striiformis*, where proteins engaged in oxidative folding were salient (Yang et al. 2016).

#### Direct defense and gene regulation

Protease inhibitors are another direct approach through which plants counteract pathogen enzymes. The fact that Trypsin inhibitor 4 (Spot 649) significantly rises in infected rose apple tissues is an indicator of an active effort to suppress the fungal proteases, which in the absence would have broken down host cell walls and tissues. Protease inhibitor up-regulation is therefore one of the significant obstacles that delay the colonization of *L. theobromae*. The same role has been reported in soybean, where improved trypsin inhibitor expression significantly decreased pathogenic fungal growth by affecting digestive processes (Sultana et al. 2023), and in the apple fruit infected with *P. expansum*, where elevated levels of protease inhibitors had a role in resistance to fungal virulence factors (Wang et al. 2024).

On the regulatory level, Telomere repeat-binding factor 1 (Spot 242), which is associated with genome stability, was found to be elevated. This observation may suggest a possible role in stress-responsive gene regulation, similar to responses reported in *Arabidopsis thaliana* under abiotic stress, although further molecular validation is required to confirm this function (Amiard et al. 2023). It was upregulated with the high expression of Alpha/beta hydrolase domain-containing protein WAV2 (Spot 283), which is normally engaged in cell wall remodelling and gravitropism in the root (Mochizuki et al. 2005). This protein could be associated with disturbed lipid signaling or cell wall reinforcement to limit fungal proliferation, as increased hydrolase activity in grapevine berries infected with *B. cinerea* in early infection (Blanco-Ulate et al. 2015).

#### Metabolic reprogramming and energy supply

Biotic stress has a high energetic cost to the plants and often causes metabolic reprogramming. It indicates increased degradation of amino acids as an alternative source of ATP via the upregulation of Probable 3-hydroxyisobutyrate dehydrogenase-like 1 (Spot 347), which is a mitochondrial enzyme (Schertl et al. 2017). Other metabolic pathways, at the same time, seem to be impaired. One of the most important enzymes in the production of benzylisoquinoline alkaloid, scoulerine-9-O-methyltransferase 1 (Spot 474) was lowered, which suggests that *L. theobromae* can inhibit the synthesis of antimicrobial alkaloid secondary metabolites. This is consistent with proteomic findings of mango infected with *L. theobromae*, which revealed the pathogen to suppress secondary metabolism of the host to encourage colonization (Munirah et al. 2017).

Due to the cost of metabolically expensive resistance to both biotrophic and necrotrophic pathogens, plants will tend to reorganize central and secondary metabolism to meet the energy requirements of immunity (Schertl et al. 2017). The up-regulation of Probable 3-hydroxyisobutyrate dehydrogenase-like 1, a mitochondrial enzyme that breaks down branched-chain amino acids (BCAA) most especially valine in this study, substantiates the hypothesis that there is energy loss in the rose apple tissues that are infected. Low carbohydrate conditions due to metabolic stress caused by infection are known to cause plants to switch to alternative respiratory substrates and oxidative phosphorylate amino acids like valine, leucine, and isoleucine to produce ATP (Peng et al. 2015). This transition is a survival mechanism whereby growth is compromised in order to divert energy towards the synthesis of antimicrobial enzymes, ROS and cell wall strengthening (Galili et al. 2014).

BCAA catabolism has also been extensively reported in plant-pathogen interactions as well as in plant-stress interactions. To illustrate, when *Arabidopsis* are subjected to prolonged periods of darkness, and thus, the mimicry of carbohydrate starvation, genes that encode BCAA-degrading enzymes towards 3-hydroxyisobutyrate are highly expressed to ensure ATP levels stay constant (Peng et al. 2015). In the same manner, a proteomic study of rice during drought stress found that enzymes of valine degradation became active to offset the diminished photosynthetic rate (Wu et al. 2016). Such activation in *L. theobromae* infection is an indication that the pathogen can use the sugar reserves of the host or establish an intense metabolic sink in the site of infection, compelling the fruit to metabolize amino acids (Morkunas and Ratajczak 2014). This is made worse by concurrent deactivation of Scoulerine-9-O-methyltransferase 1 (Spot 474) a key enzyme in the production of benzylisoquinoline alkaloid (BIA). Other BIA is berberine, sanguinarine, and scoulerine, which are synthesized de novo as phytoalexins which directly inhibit the pathogen by its antimicrobial effect (Hagel and Facchini 2013). Inhibition of scoulerine-9-O-methyltransferase is thus counterintuitive whereby one would expect more alkaloid production during infection. This paradox is probably an indication that virulence factors of *L. theobromae* were effective in the reprogramming of host metabolism to suppress the defense-related secondary metabolite pathways (Salvatore et al. 2020). In line with this, mango-*L. theobromae* interaction studies have revealed that the pathogen releases effector molecules and phytotoxins, such as jasmonic acid analogs and 3-indolecarboxylic acid, which interfere with host signaling pathways and block phenylpropanoid and alkaloid biosynthetic genes. Fruits of the mango *L. theobromae* infected with *L. theobromae* have less defensive polyphenolic compounds compared to fruits infected with *Colletotrichum gloeosporioides*, which emphasizes the special efficiency of the pathogen in overcoming the protective consequences of the chemical defense of fruits (Abd Murad et al. 2022).

#### Disruption of signaling and structural integrity

Although a number of defense layers were activated, several important signaling and structural pathways were ultimately impaired. Plant immunity largely relies on lipid signaling, especially through oxylipins like jasmonic acid, which facilitate response to *necrotrophs*. One of the enzymes that are involved in the synthesis of oxylipins is the Linoleate 9 S-lipoxygenase (Spot 506) (Marcos et al. 2015), which is downregulated in infected fruits, which may indicate that *L. theobromae* actively inhibits the host alarm system or that this process does not occur in the face of tissue decay. This trend is in line with the case of grapevine infected with *L. theobromae*, in which hormonal signaling was perturbed to allow infection (Félix et al. 2019).

Host structural defenses were also seriously weakened. Downregulation of Lanosterol synthase (Spot 445), a major enzyme in sterol biosynthesis, indicates impaired membrane repair and helps explain the observed tissue softening. Similarly, reduced expression of WAT1 protein (Spot 562), required for secondary cell wall formation and auxin transport (Ranocha et al. 2013), points to a diminished capacity to reinforce physical barriers. This structural vulnerability is exacerbated by decreased levels of 3 phosphoshikimate 1-carboxyvinyl transferase 1 (Spot 527) (EPSP synthase), a key enzyme of the shikimate pathway that supplies precursors for salicylic acid and lignin (Tall and Puigbò 2021). Inhibiting this pathway effectively deprives the plant of both chemical defenses and structural polymers. Similar downregulation of shikimate pathway enzymes and reduced lignification have been reported in banana fruit infected with *Fusarium oxysporum*, leading to increased susceptibility (Li et al. 2017).

The capability of the rose apple fruit to sense and modulate stress was also severely affected. Downregulation of Phytochrome B (Spot 609), a light receptor that interfaces with defense signaling (Kim et al. 2021), together with reduced Scoulerine-9-O-methyltransferase 1 (Spot 474), underscores the impaired capacity of the fruit to both perceive the pathogen and synthesize chemical defenses. In addition, reduced expression of F-box/FBD/LRR-repeat protein At3g51530 (Spot 488), a component of the ubiquitin-mediated protein degradation machinery, indicates that timely turnover of key regulatory proteins in defense signaling may be compromised.

### Implications of protein–protein interaction networks

STRING network analysis of the differentially expressed proteins revealed an interconnected functional network that likely underpins rose apple defense against *L. theobromae*. Central nodes in this network include Heat shock protein 90 − 1 (Hsp90-1), Heat shock 70 kDa protein (Hsp70), Peptidyl-prolyl cis-trans isomerase (PPIase), Telomere repeat-binding factor 1, and Trypsin inhibitor 4. The prominent position of Hsp90 and Hsp70 suggests that these chaperones act as key coordinators of immune responses by stabilizing Resistance (R) proteins and mediating effector recognition (Park and Seo 2015). Their interaction with PPIase, which accelerates protein folding, establishes a quality control hub that preserves the functional integrity of defense proteins under proteotoxic stress (Pogorelko et al. 2014).

**Fig. 6:**
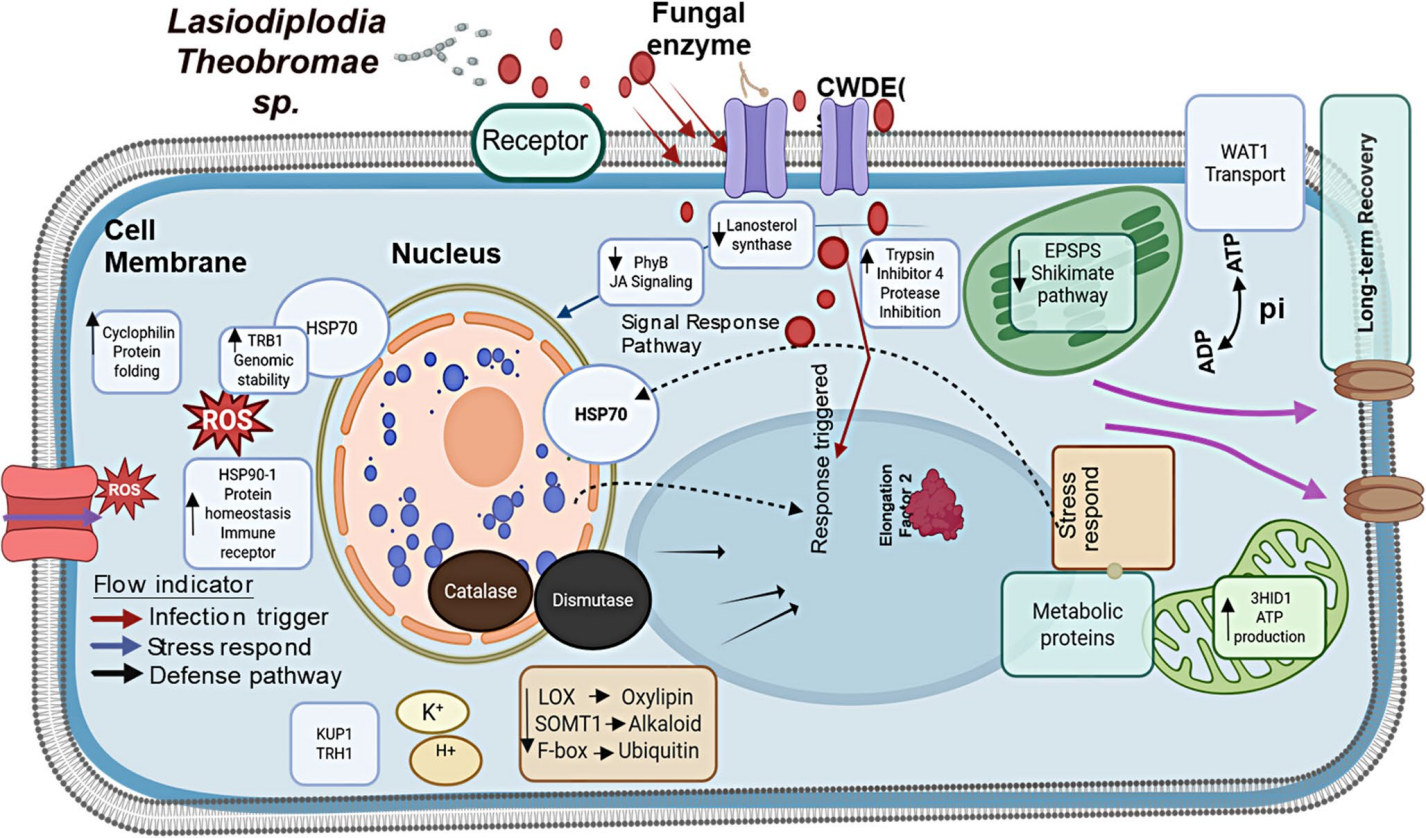
Proposed model of plant responses to *Lasiodiplodia theobromae* infection. Pathogen enzymes/cell wall–degrading enzymes (CWDEs) are perceived at the plasma membrane, triggering ROS production and signaling that involves HSP70/HSP90, TRB1, and the nucleus. Antioxidant enzymes (catalase, dismutase) and defense metabolism (LOX–oxylipin, SOMT1–alkaloid, F-box/ubiquitin) are activated, while metabolic reprogramming engages the shikimate pathway (EPSPS) and transport processes (WAT1). Mitochondrial activity (3HID1) supports ATP supply, leading to stress response and long-term recovery. Colored arrows indicate infection trigger, stress response, and defense pathways

The network also links signaling processes to physical defense mechanisms. Alpha/beta hydrolase domain-containing proteins, which are widely associated with plant development and stress responses, may play roles in cell wall reinforcement and in the regulation of hormone-mediated defense pathways (Mindrebo et al. 2016). At the same time, Trypsin inhibitor 4 reflects direct biochemical counterattack, where the host produces protease inhibitors to neutralize fungal hydrolytic enzymes targeting the cell wall (Macedo et al. 2016). Together, these interactions point to a multilayered defense strategy that integrates chaperone-mediated signaling, metabolic reprogramming, and direct antimicrobial activity. Enhancing the stability or activity of these regulatory hubs may therefore represent a promising biotechnological approach to improve resistance in rose apple and reduce postharvest losses (Fig. 6).

Under intensified stress conditions, intracellular levels of reactive oxygen species (ROS) rise markedly, leading to the activation of enzymatic antioxidant defence systems, including superoxide dismutase, catalase and ascorbate peroxidase, among others. This coordinated antioxidant response is likely supported by the action of proteins such as oxysterol-binding protein-related protein and stromal 70 kDa heat shock-related protein, which contribute to redox homeostasis and promote protein protection and stability under stress.

In addition, cytosolic- and nuclear-associated protein synthesis processes are strongly implicated, with supporting roles in mitochondria and ER plays a pivotal role in the plant’s defence. The upregulation of Heat shock protein 90 − 1 (HSP90 -1) and Heat shock 70 kD protein (HSP70) increases the ability of the plant to stabilize and fold essential immune regulators such as Resistance (R) proteins and stress -signaling elements, thus ensuring efficient pathogen recognition and signal transduction during infection induced proteotoxic stress. This is also facilitated by peptidyl-prolyl cis-trans isomerase (PPIase), which increases the appropriate folding of defence-related proteins, which allows a quick deployment of functional effectors during the initial phases of fungal attack. Telomere repeat-binding factor 1 (TRB1) assists in genomic stability as well as stress-responsive gene regulation, which supports long-term defence. In addition, the mitochondrial enzyme Probable 3-hydroxyisobutyrate dehydrogenase-like 1 that signals a change to branched-chain amino acid metabolism to provide additional energy to generate ROS, antimicrobial enzyme synthesis, and cell wall strengthening. Proteomic study of *L. theobromae* infection in rose apple, therefore, indicates a web of essential molecular actors in plant defence, which can be used in crop enhancement. By targeting proteins in the host that are upregulated, including HSP90-1 and HSP70, which stabilize immune receptors and signaling antigens, PPIase, which promotes protein folding of defence-related proteins, and 3-hydroxyisobutyrate dehydrogenase-like 1, which aids energy provision during stress, agricultural scientists could breed rose apple and other crops related to rose apple having increased resistance to fungal infections. Genetic engineering or breeding the selected proteins is one way of creating varieties that are more resistant to postharvest diseases. As an example, more active or stable expression or stabilization of HSP90, HSP70, and PPIase may enhance protein homeostasis and stress tolerance, whereas alteration of metabolic and structural regulators like WAV2 and associated hydrolases may enhance cell wall integrity and reduce the ability of pathogens to colonize cells. The proteins are good targets of interventions designed to enhance disease resistance and minimize postharvest losses in tropical fruit production.

## Conclusions and future directions

This proteomic investigation demonstrates that rose apple mounts a dynamic molecular response following fungal challenge. The increased abundance of proteins associated with ribosomal function, lipid-related processes, and redox regulation, together with the reduced accumulation of several stress-associated proteins, indicates substantial modulation of translational activity and lipid metabolic pathways during infection. Future studies using advanced quantitative proteomics and functional validation approaches will be necessary to confirm the precise roles of these proteins in the interaction between *Syzygium samarangense* and *Lasiodiplodia theobromae*. Future studies should therefore focus on clarifying the biological functions of these candidate proteins, determining how their expression can be optimally regulated, and evaluating how the coordinated balance between up- and down-regulated proteins influences overall defence performance against postharvest pathogens.

Although this study provides new insights into the proteomic response of rose apple to L. theobromae, several limitations should be acknowledged. The two-dimensional electrophoresis-based workflow used in this study has several inherent limitations. First, the analysis was conducted at a single time point after infection, which may not fully capture the dynamic temporal changes in protein expression during disease progression. In addition, the 2DE-based proteomic approach has a limited capacity to resolve low-molecular-weight and low-abundance proteins, which may have restricted the detection of important regulatory proteins involved in plant defense. Consequently, the number of proteins successfully identified remained relatively limited. This was further constrained by the absence of a fully annotated rose apple genome, which restricted accurate protein assignment. As a result, protein identification relied on homology-based searches against related plant species, which may have excluded species-specific proteins or resulted in ambiguous annotations. Finally, although several proteins associated with stress response and defense pathways were identified, functional validation experiments were not conducted, and therefore the proposed roles of these proteins in the rose apple–pathogen interaction remain to be experimentally confirmed.

It is expected that future proteomic investigations, especially following the completion of the rose apple genome sequence, will substantially improve protein coverage and annotation accuracy. Moreover, the biochemical characteristics of ripe rose apple fruit, including high water content and heterogeneous ripening stages, likely reduced protein recovery efficiency and hindered the detection of low-abundance, infection-responsive proteins. The relatively small number of biological samples also limits the broader generalisation of the present findings. Future work should therefore incorporate larger sample sets and advanced quantitative proteomic strategies, such as label-free quantification, together with independent validation approaches, including Western blot analysis, to confirm the expression patterns of the identified proteins and to strengthen the biological interpretation of the results.

## Supplementary Information

Below is the link to the electronic supplementary material.

>Supplementary figure 1

## Data Availability

No datasets were generated or analysed during the current study.

## References

[CR1] Abd Murad N, Mustafa M, Shaari K, Mohd Zainudin NJLAM (2022) Micrograph analysis of morphological alteration and cellular damage of fruit rot fungal pathogens treated with Averrhoa bilimbi fruit and Garcinia mangostana pericarp ethanolic extracts. Lett Appl Microbiol 75(5):1319–1329 35934942 10.1111/lam.13801

[CR3] Al-Obaidi JR, Hussin SNIS, Saidi NB, Rahmad N, Idris AS (2017) Comparative proteomic analysis of Ganoderma species during in vitro interaction with oil palm root. Physiol Mol Plant Pathol 99:16–24. 10.1016/j.pmpp.2017.02.001

[CR2] Al-Obaidi J, Rahmad N, Hanafi N (2023) *Aspergillus niger* causing fruit rot disease on rose apple in Malaysia. New Disease Rep 48:1–2. 10.1002/ndr2.12216

[CR4] Amiard S, Feit L, Simon L, Goff SL, Loizeau L, Wolff L, Butter F, Bourbousse C, Barneche F, Tatout C, Probst AV (2023) Distinct clades of TELOMERE REPEAT BINDING transcriptional regulators interplay to regulate plant development. Cold Spring Harbor Lab 1–61. 10.1101/2023.08.16.553498

[CR5] Aslam B, Basit M, Nisar M, Khurshid MA, Rasool MH (2017) Proteomics: Technologies and their applications. J Chromatogr Sci 55(2):182–196. 10.1093/chromsci/bmw167 28087761 10.1093/chromsci/bmw167

[CR6] Banadka A, Wudali NS, Al-Khayri JM, Nagella P (2022) The role of *Syzygium samarangense* in nutrition and economy: An overview. South Afr J Bot 145:481–492. 10.1016/j.sajb.2022.03.014

[CR7] Bano A, Gupta A, Prusty MR, Kumar M (2023) Elicitation of fruit fungi infection and its protective response to improve the postharvest quality of fruits. Stresses 3(1):231–255. 10.3390/stresses3010018

[CR8] Blanco-Ulate B, Amrine KCH, Collins TS, Rivero RM, Vicente AR, Morales-Cruz A, Doyle CL, Ye Z, Allen G, Heymann H, Ebeler SE, Cantu D (2015) Developmental and metabolic plasticity of white-skinned grape berries in response to *Botrytis cinerea* during noble rot. Plant Physiol 169(4):2422–2443. 10.1104/pp.15.00852 10.1104/pp.15.00852 PMC4677888 26450706

[CR9] Chan Z (2012) Proteomic responses of fruits to environmental stresses. Front Plant Sci 3:311. 10.3389/fpls.2012.00311 23335934 10.3389/fpls.2012.00311 PMC3541545

[CR10] Chandramouli K, Qian PY (2009) Proteomics: challenges, techniques and possibilities to overcome biological sample complexity. Hum genomics proteomics: HGP 2009:1–22. 10.4061/2009/239204 10.4061/2009/239204 PMC2950283 20948568

[CR12] Chen Y, Lan X, He R, Wang M, Zhang Y, Yang Y (2024) Biological characteristics, pathogenicity, and sensitivity to fungicides of four species of Lasiodiplodia on avocado fruits. Horticulturae 10(11):1190

[CR11] Chen Y, Hu A, Zhang L, Xu Q, Ma X, Liu H, Yin J (2026) *Bacillus velezensis* P15 combats mango postharvest rot caused by *Lasiodiplodia theobromae* via disruption of fungal energy metabolism. Food Control 185:112051. 10.1016/j.foodcont.2026.112051

[CR13] Cipriano AK, Gondim DM, Vasconcelos IM, Martins JA, Moura AA, Moreno FB, Monteiro-Moreira AC, Melo JG, Cardoso JE, Paiva AL, Oliveira JT (2015) Proteomic analysis of responsive stem proteins of resistant and susceptible cashew plants after *Lasiodiplodia theobromae* infection. J Proteom 113:90–109. 10.1016/j.jprot.2014.09.022 10.1016/j.jprot.2014.09.022 25289588

[CR14] Domingo G, Locato V, Cimini S, Ciceri L, Marsoni M, De Gara L, Bracale M, Vannini C (2024) A comprehensive characterization and expression profiling of defensin family peptides in *Arabidopsis thaliana* with a focus on their abiotic stress-specific transcriptional modulation. Curr Plant Biology 39:100376. 10.1016/j.cpb.2024.100376

[CR15] Esua JO, Chin NL, Yusof YA, Sukor R (2017) Antioxidant Bioactive Compounds and Spoilage Microorganisms of Wax Apple (*Syzygium samarangense*) during Room Temperature Storage. Int J Fruit Sci 17(2):188–201. 10.1080/15538362.2017.1285263

[CR16] Félix C, Meneses R, Gonçalves MFM, Tilleman L, Duarte AS, Jorrín-Novo JV, Van de Peer Y, Deforce D, Van Nieuwerburgh F, Esteves AC, Alves A (2019) A multi-omics analysis of the grapevine pathogen *Lasiodiplodia theobromae* reveals that temperature affects the expression of virulence- and pathogenicity-related genes. Sci Rep 9(1):13144. 10.1038/s41598-019-49551-w 31511626 10.1038/s41598-019-49551-w PMC6739476

[CR17] Fenta L, Mekonnen H, Kabtimer N (2023) The Exploitation of Microbial Antagonists against Postharvest Plant Pathogens. Microorganisms 11(4):1–17. 10.3390/microorganisms11041044 10.3390/microorganisms11041044 PMC10143894 37110467

[CR18] Galili G, Avin-Wittenberg T, Angelovici R, Fernie AR (2014) The role of photosynthesis and amino acid metabolism in the energy status during seed development. Front Plant Sci 5:447. 10.3389/fpls.2014.00447 25232362 10.3389/fpls.2014.00447 PMC4153028

[CR19] Gao L, Wang Y, Li Z, Zhang H, Ye J, Li GJF (2016) Gene expression changes during the gummosis development of peach shoots in response to *Lasiodiplodia theobromae* infection using RNA-Seq. Front Physiol 7:170 10.3389/fphys.2016.00170 PMC4861008 27242544

[CR20] Gobena S, Admassu B, Kinde MZ, Gessese AT (2024) Proteomics and its current application in biomedical area: Concise review. The Scientific World J 2024(4454744):13 10.1155/2024/4454744 10.1155/2024/4454744 PMC10894052 38404932

[CR21] Hagel JM, Facchini PJ (2013) Benzylisoquinoline alkaloid metabolism: a century of discovery and a brave new world. Plant Cell Physiol 54(5):647–672. 10.1093/pcp/pct020 23385146 10.1093/pcp/pct020

[CR22] Han Z, Schneiter RJFPS (2024) Dual functionality of pathogenesis-related proteins: defensive role in plants versus immunosuppressive role in pathogens. Front Plant Sci 15:1368467 10.3389/fpls.2024.1368467 PMC11327054 39157512

[CR23] He C, Lin J, Wu H, Zheng J, Zhang Y, Zhang Y, Li Z, Liang Y, Lu Y, Yi K, Wu W (2025) Morphological and molecular characterization of *Lasiodiplodia theobromae* causing stem gummosis disease in rubber trees and its chemical control strategies. Microorganisms 13(7):1–12 10.3390/microorganisms13071586 10.3390/microorganisms13071586 PMC12299842 40732095

[CR24] Jian Y, Gong D, Wang Z, Liu L, He J, Han X, Tsuda K (2024) How plants manage pathogen infection. EMBO Rep 25(1):31–44. 10.1038/s44319-023-00023-3 38177909 10.1038/s44319-023-00023-3 PMC10897293

[CR25] Jiang Y, Rex DAB, Schuster D, Neely BA, Rosano GL, Volkmar N, Momenzadeh A, Peters-Clarke TM, Egbert SB, Kreimer S, Doud EH, Crook OM, Yadav AK, Vanuopadath M, Hegeman AD, Mayta ML, Duboff AG, Riley NM, Moritz RL, Meyer JG (2024) Comprehensive Overview of Bottom-Up Proteomics Using Mass Spectrometry. ACS Meas Sci Au 4(4):338–417. 10.1021/acsmeasuresciau.3c00068 39193565 10.1021/acsmeasuresciau.3c00068 PMC11348894

[CR26] Jiang Y, Wang J, Sun A, Zhang H, Yu X, Qin W, Ying W, Li Y, Chang C, Wang X, Xie L, Liu W, Liu J, Zhang X, Yan Q, Zou Y, Zhao C, Sun H, Zhang J, Su S, Gao Q, He F (2025) The coming era of proteomics-driven precision medicine. Natl Sci Rev 12(8):1–15. 10.1093/nsr/nwaf278 10.1093/nsr/nwaf278 PMC12365760 40842868

[CR27] Kaur G, Singh S, Singh H, Chawla M, Dutta T, Kaur H, Bender K, Snedden WA, Kapoor S, Pareek A, Singh P (2015) Characterization of Peptidyl-Prolyl Cis-Trans Isomerase- and Calmodulin-Binding Activity of a Cytosolic *Arabidopsis thaliana* Cyclophilin AtCyp19-3. PLoS ONE 10(8):e0136692. 10.1371/journal.pone.0136692 26317213 10.1371/journal.pone.0136692 PMC4552658

[CR28] Khan M.N., Allawi M.Y., Adnan B.B., Ahmad N.A., Sinang S.C., Mohd Noor N.N., Rahman M.A., Kamaruddin N., Rahmad N., Khairuddin R.F.R., Al-Obaidi J.R. (2025) Molecular detection and symptomatology of *Lasiodiplodia theobromae* (Ascomycota) infecting rose apples (*Syzygium samarangense*) in Malaysia. Ukrainian Bot J 82(5):453–459

[CR29] Kim JY, Lee J-H, Park C-M (2021) A multifaceted action of phytochrome B in plant environmental adaptation. Front Plant Sci 12:1–8. 10.3389/fpls.2021.659712 10.3389/fpls.2021.659712 PMC8258378 34239522

[CR31] Li W, Li C, Sun J, Peng M (2017) Metabolomic, Biochemical, and Gene Expression Analyses Reveal the Underlying Responses of Resistant and Susceptible Banana Species during Early Infection with *Fusarium oxysporum* f. sp. cubense. Plant Dis 101(4):534–543. 10.1094/pdis-09-16-1245-re 30677364 10.1094/PDIS-09-16-1245-RE

[CR30] Li R, Yao J, Ming Y, Guo J, Deng J, Liu D, Li Z, Cheng Y (2023) Integrated proteomic analysis reveals interactions between phosphorylation and ubiquitination in rose response to *Botrytis infection*. Hortic Res 11(1):1–14. 10.1093/hr/uhad238 10.1093/hr/uhad238 PMC10782497 38222823

[CR32] Macedo ML, Ribeiro SF, Taveira GB, Gomes VM, de Barros KM, Maria-Neto S (2016) Antimicrobial Activity of ILTI, a Kunitz-Type Trypsin Inhibitor from *Inga laurina* (SW.) Willd. Curr Microbiol 72(5):538–544. 10.1007/s00284-015-0970-z 26769111 10.1007/s00284-015-0970-z

[CR33] Majumder S, Acharyya S, Ghosh A, Chakraborty S, Sarkar S, Saha S, Bhattacharya M (2021) Insights into low biological activity of wax apple (*Syzygium samarangense*) juice by in vitro phytochemical investigation with special reference to metabolomics. Biofarmasi J Nat Prod Biochem 19:30–38. 10.13057/biofar/f190106

[CR34] Marcos R, Izquierdo Y, Vellosillo T, Kulasekaran S, Cascón T, Hamberg M, Castresana C (2015) 9-Lipoxygenase-Derived Oxylipins Activate Brassinosteroid Signaling to Promote Cell Wall-Based Defense and Limit Pathogen Infection. Plant Physiol 169(3):2324–2334. 10.1104/pp.15.00992 26417008 10.1104/pp.15.00992 PMC4634075

[CR35] Meng C-R, Zhang Q, Yang Z-F, Geng K, Zeng X-Y, Chethana KT, Wang Y (2021) Lasiodiplodia syzygii sp. nov.(Botryosphaeriaceae) causing post-harvest water-soaked brown lesions on *Syzygium samarangense* in Chiang Rai, Thailand. Biodivers data J 9:e60604. 10.3897/BDJ.9.e60604 33510578 10.3897/BDJ.9.e60604 PMC7809010

[CR36] Mindrebo JT, Nartey CM, Seto Y, Burkart MD, Noel JP (2016) Unveiling the functional diversity of the alpha/beta hydrolase superfamily in the plant kingdom. Curr Opin Struct Biol 41:233–246. 10.1016/j.sbi.2016.08.005 27662376 10.1016/j.sbi.2016.08.005 PMC5687975

[CR37] Mochizuki S, Harada A, Inada S, Sugimoto K, Stacey N, Wada T, Ishiguro S, Okada K, Sakai T (2005) The Arabidopsis WAVY GROWTH 2 protein modulates root bending in response to environmental stimuli. Plant Cell 17:537–547. 10.1105/tpc.104.028530 15659627 10.1105/tpc.104.028530 PMC548824

[CR38] Mohapatra S, Rithesh L, Tiwari RK, Behera L, Lal MK, Kumar R (2025) Plant defense mechanisms: Innate and induced immunity in plants. 221–234. 10.1079/9781800627659.0015

[CR39] Morkunas I, Ratajczak LJAPP (2014) The role of sugar signaling in plant defense responses against fungal pathogens. Acta Physiologiae Plant 36(7):1607–1619

[CR40] Mukaromah A (2020) Wax Apple (*Syzygium samarangense* (Blume) Merr. & L.M. Perry): A Comprehensive Review in Phytochemical and Physiological Perspectives. Al-Hayat: J Biology Appl Biology 3(40):40–58. 10.21580/ah.v3i1.6070

[CR41] Munirah M, Azmi A, Yong S, Nur Ain Izzati MJPPQ (2017) Characterization of *Lasiodiplodia theobromae* and *L. pseudotheobromae* causing fruit rot on pre-harvest mango in Malaysia. Plant Pathol Quarantine 7(2):202–213

[CR42] Ortea I, O’Connor G, Maquet A (2016) Review on proteomics for food authentication. J Proteom 147:212–225. 10.1016/j.jprot.2016.06.033 10.1016/j.jprot.2016.06.033 27389853

[CR43] Papavasileiou A, Tanou G, Samaras A, Samiotaki M, Molassiotis A, Karaoglanidis G (2020) Proteomic analysis upon peach fruit infection with *Monilinia fructicola* and *M. laxa* identify responses contributing to brown rot resistance. Sci Rep 10(1):7807. 10.1038/s41598-020-64864-x 32385387 10.1038/s41598-020-64864-x PMC7210933

[CR44] Park CJ, Seo YS (2015) Heat Shock Proteins: A Review of the Molecular Chaperones for Plant Immunity. plant Pathol J 31(4):323–333. 10.5423/ppj.Rw.08.2015.0150 26676169 10.5423/PPJ.RW.08.2015.0150 PMC4677741

[CR45] Peng C, Uygun S, Shiu SH, Last RL (2015) The Impact of the Branched-Chain Ketoacid Dehydrogenase Complex on Amino Acid Homeostasis in Arabidopsis. Plant Physiol 169(3):1807–1820. 10.1104/pp.15.00461 25986129 10.1104/pp.15.00461 PMC4634046

[CR46] Peng J, Li X, Li Y, Zhang W, Zhou Y, Yan J (2022) *Lasiodiplodia theobromae* protein LtScp1 contributes to fungal virulence and protects fungal mycelia against hydrolysis by grapevine chitinase. Environ Microbiol 24(10):4670–4683. 10.1111/1462-2920.16155 36054544 10.1111/1462-2920.16155 PMC9804331

[CR47] Pogorelko GV, Mokryakova M, Fursova OV, Abdeeva I, Piruzian ES, Bruskin SA (2014) Characterization of three *Arabidopsis thaliana* immunophilin genes involved in the plant defense response against Pseudomonas syringae. Gene 538(1):12–22. 10.1016/j.gene.2014.01.029 24440291 10.1016/j.gene.2014.01.029

[CR48] Ranocha P, Dima O, Nagy R, Felten J, Corratgé-Faillie C, Novák O, Morreel K, Lacombe B, Martinez Y, Pfrunder S, Jin X, Renou J-P, Thibaud J-B, Ljung K, Fischer U, Martinoia E, Boerjan W, Goffner D (2013) Arabidopsis WAT1 is a vacuolar auxin transport facilitator required for auxin homoeostasis. Nat Commun 4(1):2625. 10.1038/ncomms3625 24129639 10.1038/ncomms3625 PMC3826630

[CR49] Salem SS, Elsayed HE, Shabana S, Khazaal MT, Moharram FA (2023) Phytochemical profile and antimicrobial activity of essential oils from two *Syzygium* species against selected oral pathogens. BMC Complement Med Ther 23(1):448. 10.1186/s12906-023-04277-1 38087292 10.1186/s12906-023-04277-1 PMC10714517

[CR50] Salvatore MM, Alves A, Andolfi A (2020) Secondary Metabolites of *Lasiodiplodia theobromae*: Distribution, Chemical Diversity, Bioactivity, and Implications of Their Occurrence. Toxins 12(7):1–29. 10.3390/toxins12070457 10.3390/toxins12070457 PMC7405015 32709023

[CR51] Saroj P, Shah N (2023) Syzygium samarangense (Jamb) -Its Ethnobotanical Knowledge, Phytochemical Studies, Pharmacological Aspects and Future Prospects 2022.

[CR52] Schertl P, Danne L, Braun HP (2017) 3-Hydroxyisobutyrate Dehydrogenase Is Involved in Both, Valine and Isoleucine Degradation in *Arabidopsis thaliana*. Plant Physiol 175(1):51–61. 10.1104/pp.17.00649 28705827 10.1104/pp.17.00649 PMC5580760

[CR53] Subbulakshmi, K., Satish, S., & Shabaraya, A. R. (2021). Rose apple fruit: A pharmacological review. World Journal of Pharmacy and Pharmaceutical Sciences 10(4):842–849

[CR54] Shah P, Powell AL, Orlando R, Bergmann C, Gutierrez-Sanchez G (2012) Proteomic analysis of ripening tomato fruit infected by *Botrytis cinerea*. J Proteome Res 11(4):2178–2192. 10.1021/pr200965c 22364583 10.1021/pr200965c PMC4874510

[CR55] Singh D, Sharma RR (2018) Postharvest Diseases of Fruits and Vegetables and Their Management. In. pp 1–52. 10.1016/B978-0-12-812698-1.00001-7

[CR56] Sui Y, Li Z, Xiao X, Deng W, Duan B (2025) The role of induced resistance in mitigating postharvest fungal diseases in fruits and vegetables. J Adv Res Article in Press:1–21. 10.1016/j.jare.2025.08.037 10.1016/j.jare.2025.08.037 PMC13131510 40850677

[CR57] Sultana MS, Mazarei M, Jurat-Fuentes JL, Hewezi T, Millwood RJ, Stewart CN (2023) Overexpression of soybean trypsin inhibitor genes decreases defoliation by corn earworm (*Helicoverpa zea*) in soybean (*Glycine max*) and *Arabidopsis thaliana*. Front Plant Sci 14:1–16. 10.3389/fpls.2023.1129454 10.3389/fpls.2023.1129454 PMC9982021 36875574

[CR58] Tall T, Puigbò P (2021) The glyphosate target enzyme 5-Enolpyruvyl Shikimate 3-phosphate synthase (EPSPS) contains several EPSPS-associated domains in fungi. Proceedings 76(1):6

[CR59] Uranga CC, Ghassemian M, Hernández-Martínez R (2017) Data from proteome analysis of *Lasiodiplodia theobromae* (Botryosphaeriaceae). Data Brief 13:124–128. 10.1016/j.dib.2017.04.058 10.1016/j.dib.2017.04.058 PMC5447515 28580409

[CR60] Wang K, Wang H, Xu M, Ngea GLN, Zhang H (2024) The proteome of *Penicillium expansum* during infection of postharvest apple is revealed using Label-Free and Parallel Reaction Monitoring(PRM)Techniques. J Proteom 298:105142. 10.1016/j.jprot.2024.105142 10.1016/j.jprot.2024.105142 38428586

[CR61] Wei Y, Zhu B, Liu W, Cheng X, Lin D, He C, Shi H (2021) Heat shock protein 90 co-chaperone modules fine-tune the antagonistic interaction between salicylic acid and auxin biosynthesis in cassava. Cell Rep 34(5):108717. 10.1016/j.celrep.2021.108717 33535044 10.1016/j.celrep.2021.108717

[CR62] Wu Y, Mirzaei M, Pascovici D, Chick JM, Atwell BJ, Haynes PA (2016) Quantitative proteomic analysis of two different rice varieties reveals that drought tolerance is correlated with reduced abundance of photosynthetic machinery and increased abundance of ClpD1 protease. J Proteom 143:73–82. 10.1016/j.jprot.2016.05.014 10.1016/j.jprot.2016.05.014 27195813

[CR63] Xing Q, Zhou X, Cao Y, Peng J, Zhang W, Wang X, Wu J, Li X, Yan J (2023) The woody plant-degrading pathogen *Lasiodiplodia theobromae* effector LtCre1 targets the grapevine sugar-signaling protein VvRHIP1 to suppress host immunity. J Exp Bot 74(8):2768–2785. 10.1093/jxb/erad055 36788641 10.1093/jxb/erad055 PMC10112684

[CR64] Xu M, Wang K, Li J, Tan Z, Godana EA, Zhang H (2022) Proteomic Analysis of Apple Response to *Penicillium expansum* Infection Based on Label-Free and Parallel Reaction Monitoring Techniques. J fungi (Basel Switzerland) 8(12):1–15. 10.3390/jof8121273 10.3390/jof8121273 PMC9780870 36547606

[CR65] Yang Y, Yu Y, Bi C, Kang Z (2016) Quantitative Proteomics Reveals the Defense Response of Wheat against *Puccinia striiformis* f. sp. tritici. Sci Rep 6(1):34261. 10.1038/srep34261 27678307 10.1038/srep34261 PMC5039691

[CR66] Zhang H, Zhao Y, Zhu J-K (2020) Thriving under Stress: How Plants Balance Growth and the Stress Response. Dev Cell 55(5):529–543. 10.1016/j.devcel.2020.10.012 33290694 10.1016/j.devcel.2020.10.012

[CR67] Zhang WJ, Zhou Y, Zhang Y, Su YH, Xu T (2023) Protein phosphorylation: A molecular switch in plant signaling. Cell Rep 42(7):112729. 10.1016/j.celrep.2023.112729 37405922 10.1016/j.celrep.2023.112729

